# Bioactive Peptides from Soft-Shelled Turtle: Extraction, Preparation and Biological Activities

**DOI:** 10.3390/foods15162773

**Published:** 2026-08-07

**Authors:** Xin Zhang, Qiuyue Hu, Yafang Shen, Xinru Liu, Jiangqi Wang, Yanna Cui, Fei Jia, Guijie Hao, Jianrong Jin

**Affiliations:** 1 College of Food Science and Engineering, Ocean University of China, Qingdao 266003, China; zx070911@163.com (X.Z.); feijia@ouc.edu.cn (F.J.); 2 Key Laboratory of Healthy Freshwater Aquaculture, Ministry of Agriculture and Rural Affairs, Huzhou Key Laboratory of Aquatic Product Quality Improvement and Processing Technology, Zhejiang Institute of Freshwater Fisheries (Zhejiang Freshwater Fishery Environmental Monitoring Station), Huzhou 313001, China; chenyang10140021@163.com (Q.H.); shenyafang1994@126.com (Y.S.); mz220230823@stu.yzu.edu.cn (X.L.); jiangqiwang@163.com (J.W.); 15967216452@163.com (Y.C.); 3 Hangzhou Haolin Agricultural Development Co., Ltd., Hangzhou 311522, China

**Keywords:** soft-shelled turtle peptide, separation and purification, functional activity, molecular mechanism, sustainability

## Abstract

**Background:** Soft-shelled turtle (*Pelodiscus sinensis*), traditionally used for both medicinal and culinary purposes, is rich in high-quality protein and amino acids, making it an excellent raw material for the preparation of bioactive peptides. In recent years, soft-shelled turtle peptides have gradually become a research focus due to their significant physiological functions, including antioxidant, immunomodulatory, and anti-fatigue activities. **Methods:** This review systematically sorts out and analyzes 76 relevant research papers published from 2002 to 2026 and comprehensively elaborates on the current state of soft-shelled turtle peptide research, encompassing sources, preparation, and purification strategies, while placing special emphasis on the diverse functional activities and the molecular mechanisms underlying the observed bioactivities. **Results:** For preparation, enzymatic hydrolysis has become the mainstream method due to its high efficiency and mild conditions; fermentation offers the advantages of low cost and the ability to simultaneously remove odours and enhance flavour, demonstrating good application potential. This review summarizes five key bioactivities, including antioxidant, anti-fatigue, antihypertensive, antitumor, and immunomodulatory effects, and specifically focusing on the current evidence from in vitro and animal studies. Antioxidant and antihypertensive activities have been validated in vivo, whereas the rest remain only tested in vitro, highlighting the need for deeper mechanistic investigation. **Conclusions:** The review provides an in-depth analysis of the molecular mechanisms through which soft-shelled turtle peptides exert their physiological activities, including free radical scavenging and the regulation of signaling pathways. This review highlights the potential of soft-shelled turtles as a sustainable source of bioactive peptides and offers strategic recommendations for future research.

## 1. Introduction

Soft-shelled turtle, also referred to as the water turtle, is native to East Asia, with a natural range spanning China, Japan, Korea, northern Vietnam, and the Russian Far East. Through anthropogenic introduction, feral populations have now been established globally [1]. Valued as a functional food, its muscle is rich in protein (16.62–17.36 g/100 g wet weight) and extremely low in fat (0.83–0.95 g/100 g wet weight), with essential amino acids accounting for approximately 40% of total amino acids, a nutritional value superior to the FAO/WHO recommended standards [2]. In addition to its nutritional value, soft-shelled turtle protein has been associated with several biological functions, including antioxidant, immunomodulatory, anti-fatigue, and mineral absorption-promoting effects [3]. Economically, *P. sinensis* is one of the most important freshwater aquaculture reptiles. China alone produces over 490,000 metric tons annually, representing a multi-billion-dollar industry, and substantial production also occurs in other Asian countries [4]. Consequently, soft-shelled turtles are not only a nutritious food source for humans but also a protein resource rich in high-quality bioactive peptides.

Bioactive peptides (BAPs) typically consist of 2 to 20 amino acids and possess a variety of physiological functions [5]. After protein intake, under the catalytic action of digestive enzymes, proteins are first broken down into small peptide molecules before being absorbed and utilized by the body. Compared to individual amino acids, BAPs are more readily absorbed by the body [6]. Bioactive peptides derived from soft-shelled turtles are produced using soft-shelled turtle muscle or processing by-products as raw materials. After pre-treatment to remove impurities, the turtle protein undergoes targeted hydrolysis using enzymatic hydrolysis or probiotic fermentation technology, yielding small-molecule peptides [7,8]. The application of soft-shelled turtle peptides can effectively overcome technical bottlenecks such as the poor solubility of soft-shelled turtle protein during food R&D, and the development of functional health foods based on these peptides shows extremely promising market prospects [9].

Despite existing reviews having covered the nutritional value and biological functions of soft-shelled turtle products, they predominantly focus on the overall characteristics of muscle tissue, while research on bioactive peptides from other tissues and processing by-products remains scattered and unsystematic. There is still a lack of comprehensive reviews that integrate the sources, preparation processes, functional activities and molecular mechanisms of soft-shelled turtle peptides across different tissues. Furthermore, basic research findings are rarely linked to industrial production practice, which provides insufficient support for the high-value utilization of processing by-products and the optimization of large-scale preparation processes.

This article reviews the sources of soft-shelled turtle peptides, introduces preparation methods using soft-shelled turtles and their processing by-products as raw materials, and summarizes research progress in their high-value utilization. It aims to promote the development of soft-shelled turtle peptide products with diverse functional properties. Through technological innovation and process optimization, the high-value utilization of soft-shelled turtle processing by-products can be achieved, and the preparation process of soft-shelled turtle peptides can be further improved. In addition, this review summarizes the functional activities and molecular mechanisms of soft-shelled turtle peptides, providing a theoretical basis for the in-depth development of soft-shelled turtles and their by-products, and laying a foundation for the healthy and sustainable development of the soft-shelled turtle industry.

## 2. Sources of Soft-Shelled Turtle Peptides

As a high-protein, low-fat aquatic resource, the edible parts of soft-shelled turtles primarily include muscle and calipash meat. In China, the soft-shelled turtle is one of the aquaculture species with high economic value; according to the 2024 China Fisheries Statistical Yearbook, the national soft-shelled turtle production volume exceeded 490,000 tonnes [4]. Traditionally, soft-shelled turtles have been used in their entirety or in cut-up form for culinary and tonic purposes; however, the substantial by-products generated during processing (such as heads, feet, bones and internal organs) are often discarded, resulting in a waste of resources [10]. In recent years, with the advancement of research into bioactive peptides, the value of these by-products has been recognized. Research has revealed significant differences in protein content and amino acid composition across various parts of the soft-shelled turtle. The dry powder protein content of the shell and calipash is 54.18%, while that of the bones and meat is 80.38%. Regarding essential amino acid composition, after proteolytic hydrolysis of the bones and meat, the total essential amino acid content reaches 39.81% [11]. Differences in protein content and amino acid composition result in distinct functional activities among the peptides hydrolyzed from different parts. This section summarizes recent research progress on the extraction of soft-shelled turtle peptides from various parts (Table 1). As shown in the table, these hydrolyzed peptides possess biological activity.

### 2.1. Bioactive Peptides Extracted from Soft-Shelled Turtle Muscle

Soft-shelled turtle muscle accounts for 40–50% of the animal’s total body weight and is the primary edible part. Soft-shelled turtle muscle is characterized by high protein and low fat content; in the amino acid composition of the muscle protein, glycine, proline and glutamic acid are present in relatively high concentrations [17]. Due to its richness in hydrophobic and acidic amino acids, soft-shelled turtle muscle protein readily releases bioactive oligopeptide fragments during enzymatic hydrolysis, demonstrating good enzymatic hydrolysis potential and good potential for enzymatic hydrolysis and fermentation. Liao et al. [18] found, through computer simulation, that after enzymatic hydrolysis with papain and bromelain, soft-shelled turtle muscle protein could generate hydrolysed peptides with antioxidant and antihypertensive activities. These findings suggest that soft-shelled turtle muscle has considerable potential as a source of bioactive peptides.

### 2.2. Bioactive Peptides Extracted from Soft-Shelled Turtle Calipash

**Figure 1:**
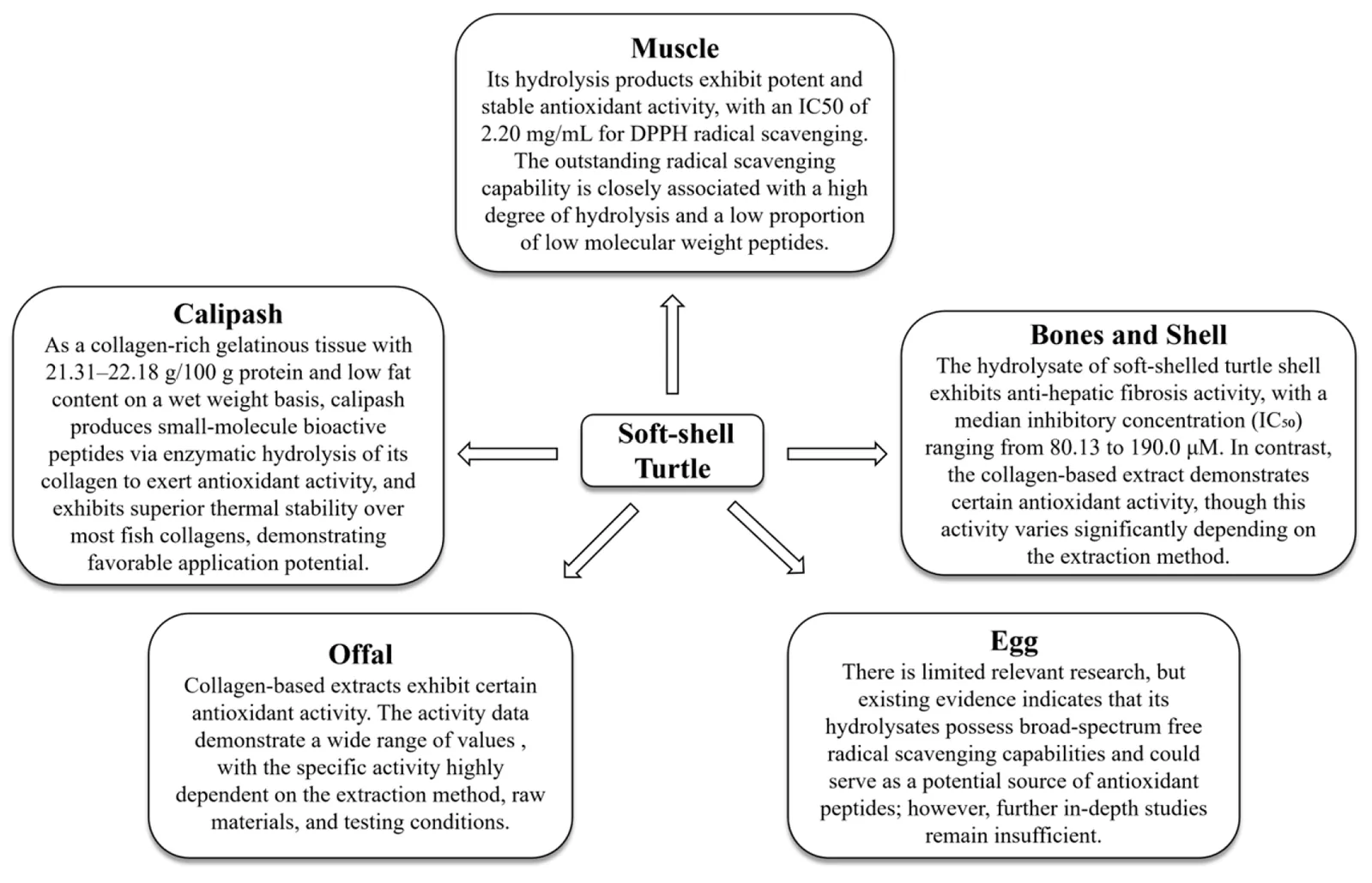
Schematic diagram of the analysis of various parts of the soft-shelled turtle (data in the chart summarized from Table 1).

The soft calipash of the soft-shelled turtle is the gelatinous connective tissue found at the edge of the carapace of the Chinese soft-shelled turtle (*Pelodiscus sinensis*), and it is also the part of the whole turtle with the highest collagen content. Studies have shown that the protein content of soft-shelled turtle calipash can reach 21.31–22.18 g/100 g wet weight (Figure 1), whilst the fat content is only 0.22–0.25 g/100 g wet weight, making it a typical low-fat, high-protein raw material [2]. In addition to basic nutrients, calipash is also rich in collagen-specific amino acids and trace elements, making it a high-quality biomaterial and functional food ingredient. As collagen in traditional biomaterials is derived from terrestrial animals such as pigs and cattle, it is susceptible to swine fever virus, BSE and foot-and-mouth disease [19]. Consequently, identifying a safe and reliable new source for collagen extraction is of paramount importance. In contrast, soft-shell turtle calipash, as an emerging source of aquatic collagen, not only offers superior biosafety but also demonstrates greater thermal stability in biomedical applications. Research by Yang et al. indicates that the denaturation temperature (Td) of soft-shelled turtle calipash collagen is 35.1 °C, whilst its melting temperature (Tm) reaches 105.14 °C, significantly higher than that of most fish collagens (15.6–32.6 °C). This confers superior structural stability at body temperature, enabling it to better meet the dual requirements of heat-resistant processing and in vivo durability for biomedical materials [20]. This structural stability at body temperature indicates its potential for heat-resistant processing and biomedical durability. In vitro simulation experiments further showed that collagen extracted from soft-shelled turtle calipash can generate small-molecule bioactive peptides and exhibit antioxidant activity. Based on its nutritional value and composition, soft-shelled turtle calipash is an ideal raw material for collagen extraction.

### 2.3. Bioactive Peptides Extracted from Soft-Shelled Turtle Offal

The offal accounts for approximately 10% to 15% of a soft-shelled turtle’s total body weight and is one of the main by-products of soft-shelled turtle processing. Soft-shelled turtle offal includes organs such as the liver, intestines and heart; the liver, in particular, is not only rich in protein but also abundant in trace elements essential to the human body, such as zinc, manganese and selenium, and thus possesses high nutritional value. Furthermore, the antioxidant peptides LEAP-1 (also known as hepatidin) and LEAP-2, which are expressed exclusively in soft-shelled turtle livers, have been reported to possess antibacterial and antiviral activities [21]. In recent years, with the rapid development of the soft-shelled turtle deep-processing industry, the efficient extraction of bioactive peptides from internal organs has become a key focus for the high-value utilisation of these resources.

### 2.4. Bioactive Peptides Extracted from Soft-Shelled Turtle Bones and Shells

Among the calcified tissues of soft-shelled turtles, the bones are considered to have the highest concentration and purity of collagen, making them a high-quality source of collagen for pharmaceutical and cosmetic applications. ACE-inhibiting peptides extractable from soft-shelled turtle bone collagen have been demonstrated to possess antihypertensive activity and can be utilized in the development of functional products for lowering blood pressure [14]. Turtle shells are the primary by-product of soft-shelled turtle processing, accounting for approximately 20–30% of the total turtle mass. Rich in collagen, polysaccharides, peptides and various minerals, they serve as a high-quality source of functional ingredients [22]. Numerous studies have been conducted on the extraction of collagen from fish [23,24]. However, aquatic collagen has poor thermal stability, which imposes significant limitations on its use. For example, collagen used in medical applications must be maintained at a temperature higher than that of the human body to prevent denaturation and loss of efficacy due to high temperatures [25]. Collagen extracted from soft-shelled turtle shells has a higher Td than that from fish sources, suggesting that collagen derived from soft-shelled turtle shells may possess superior thermal stability [20]. Furthermore, soft-shelled turtle shells are commonly used as medicinal ingredients in traditional Chinese medicine, and the oral administration of soft-shelled turtle shell powder has been shown to have anti-fatigue and anti-tumour effects [26]. However, when soft-shelled turtles are consumed as food, their shells are often discarded, leading to environmental pollution. Therefore, developing collagen from soft-shelled turtle shells not only has medicinal value, but also helps reduce resource waste.

### 2.5. Bioactive Peptides Extracted from Soft-Shelled Turtle Eggs

The total edible portion of soft-shelled turtle eggs contains 73.23% moisture, with the dry matter comprising 54.64% protein, 29.21% fat, 5.81% ash and 10.30% carbohydrates [16]. Among the 20 amino acids contained in soft-shelled turtle eggs, nine are essential amino acids that cannot be synthesised by the human body, which sufficiently demonstrates the extremely high nutritional value of soft-shelled turtle eggs. These eggs contain high levels of lipoproteins metabolism-related enzymes, transferrin protein-binding proteins and vitamin-binding proteins. After simulated gastric digestion and enzymatic hydrolysis, these proteins can release various bioactive peptides with multiple functions, including antioxidant, hypoglycaemic, antibacterial and antihypertensive activities [27]. However, given the low yield, high production costs and complex processing involved in soft-shelled turtle eggs, they are not the preferred source for the extraction of bioactive peptides.

### 2.6. Cross-Tissue Comparison of Soft-Shelled Turtle Peptide Properties

These aforementioned sources of soft-shelled turtle bioactive peptides exhibit pronounced differences in peptide yield, extraction efficiency, amino acid profile, bioactive performance, and industrial application potential (Figure 1). In terms of extraction efficiency and peptide yield, muscle tissue achieves favorable peptide recovery under conventional enzymatic hydrolysis, benefiting from its high content of soluble myofibrillar protein and well-balanced protein composition [17]. In contrast, calipash and bone tissues are dominated by insoluble collagen, which requires additional thermal denaturation or targeted pretreatment to improve enzymatic accessibility, leading to lower extraction efficiency compared with muscle tissue under identical hydrolysis conditions [28]. Offal tissues contain high levels of lipids and non-protein impurities, necessitating pre-defatting and impurity removal steps that introduce additional peptide loss during pretreatment and reduce the overall recovery rate [29]. Regarding amino acid composition, muscle tissue presents a well-balanced essential amino acid profile, with a total essential amino acid to total amino acid (TEAA/TAA) ratio of 40.72% and a total essential amino acid to total non-essential amino acid (TEAA/TNEAA) ratio of 68.68%, which meets the FAO/WHO high-quality protein standard and renders it suitable for the development of broad-spectrum nutritional peptide products [17]. Peptides derived from calipash and bone are highly enriched in glycine, proline, and hydroxyproline, showing typical collagen peptide features that endow them with functional advantages in bone health and skin repair applications [20,30]. Egg-derived peptides possess a unique proportion of branched-chain amino acids and hydrophobic amino acids, which is closely associated with their potent target-specific bioactivities [16]. With respect to bioactive properties, muscle hydrolysates exhibit a wide range of activities including antioxidant and ACE-inhibitory effects, but most of the reported activities are at moderate levels [18]. Calipash collagen peptides show favorable antioxidant activity and structural stability, while visceral hydrolysates demonstrate prominent free radical scavenging capacity [20,30]. Egg-derived peptides are distinguished by their high target specificity, with ACE-inhibitory and α-glucosidase inhibitory activities reaching micromolar IC_50_ levels, making them a promising source for screening high-activity monomeric peptides [16]. From the perspective of industrial feasibility, peptide production from muscle tissue has the most mature processing technology and stable raw material supply, but is associated with relatively high raw material costs. By-product tissues including viscera, bone, and calipash have extremely low or even negative raw material costs, representing a core direction for the high-value utilization of processing by-products; however, their industrial application is currently limited by complex pretreatment and purification processes, as well as insufficient pilot-scale production data. Notably, most existing studies focus on independent process optimization for single tissues, and systematic parallel comparisons under unified enzymatic hydrolysis and activity evaluation protocols remain scarce, which restricts the direct comparability of performance data across different tissue sources.

### 2.7. Bioactive Peptides from Different Pelodiscus sinensis Strains and Other Farmed Freshwater Turtles

Research on bioactive peptides is most comprehensive for the common cultured population of *Pelodiscus sinensis*. Over 30 monomeric peptides have been identified from multiple tissues, including representative sequences such as ARDASVLK, HNKPEVEVR, LVDGVIIR and EDYGA. Regarding molecular weight distribution, peptides below 1000 Da account for 91.38% of the total peptide mass in muscle papain hydrolysates, and these low-molecular-weight fractions exhibit marked antioxidant, ACE inhibitory, and immunomodulatory activities [9,12,16]. In contrast, studies on bioactive peptides from the grass turtle (*Chinemys reevesii*) are relatively scarce. The muscle hydrolysate similarly contains 89.23% sub-1000 Da peptides and has demonstrated antioxidant and metal-chelating properties [31]. More recently, a targeted investigation into the antiaging mechanism of Chinese pond turtle peptides identified several specific sequences, including WGDAGAE, LSEE, TVEE, and HELE, which were found to activate the Nrf2/Keap1 signaling pathway [3]. A detailed comparison of the peptide profiles from these two species is presented in Table 2.

## 3. Preparation, Separation and Purification of Soft-Shelled Turtle Peptides

**Figure 2:**
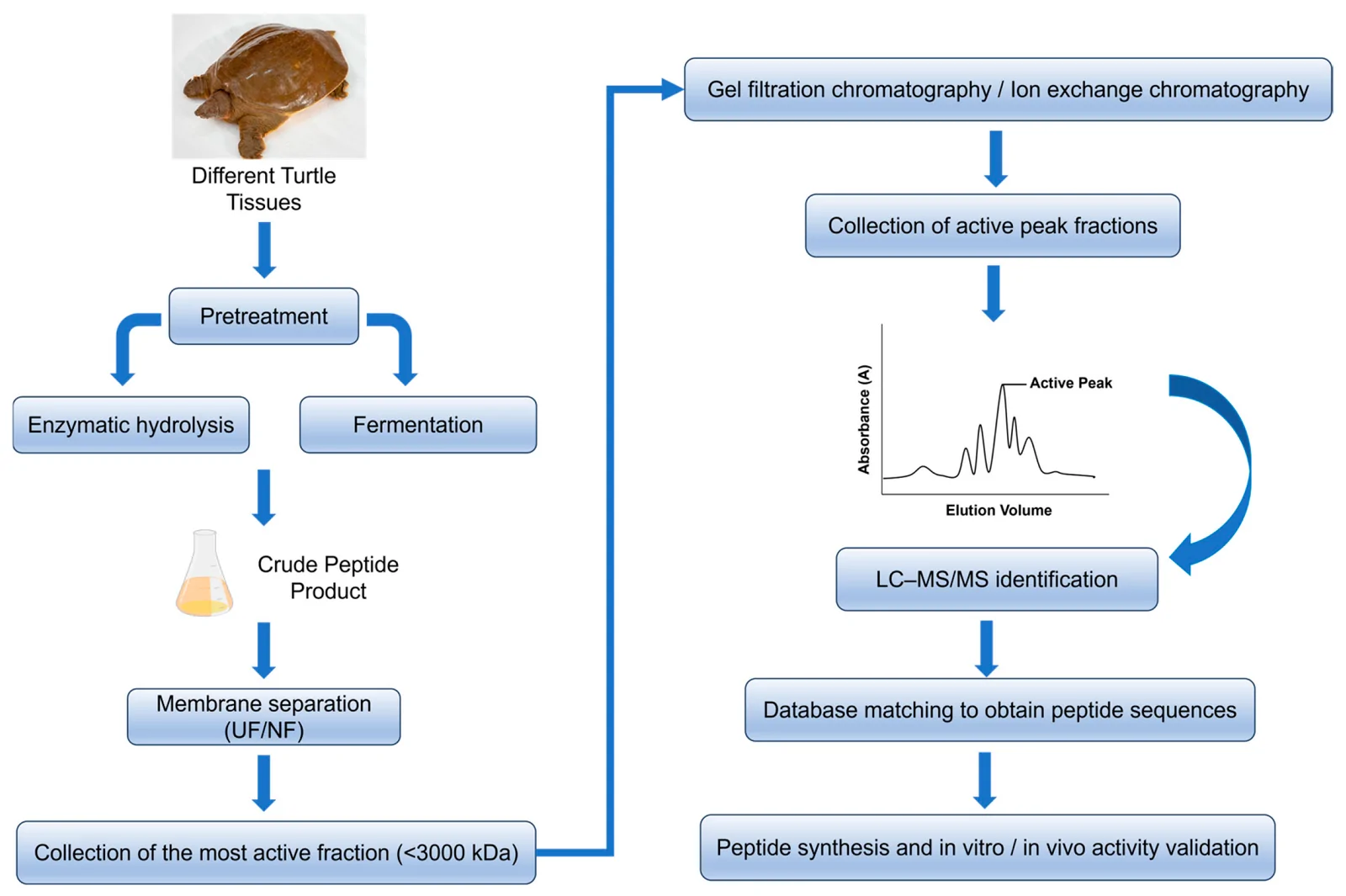
Isolation and identification workflow of soft-shelled turtle peptides.

The compositional and structural differences among soft-shelled turtle tissues directly influence the efficiency of peptide release. Muscle tissue, rich in sarcoplasmic proteins, contrasts sharply with calipash and bone, where a highly cross-linked collagen matrix predominates. Accordingly, this section systematically reviews current methods for the preparation, separation, and purification of soft-shelled turtle peptides. Emphasis is placed on how the two mainstream preparation techniques, enzymatic hydrolysis and fermentation [32], exhibit distinct tissue applicability, followed by downstream separation strategies such as membrane filtration that are essential for obtaining peptide fractions with targeted bioactivities (Figure 2).

### 3.1. Preparation Methods of Soft-Shelled Turtle Peptides

Enzymatic hydrolysis is an efficient method for preparing bioactive peptides, offering the dual advantages of high specificity and non-toxicity. This method is particularly suited for soft-shelled turtle muscle, where the relatively high sarcoplasmic protein content allows efficient enzyme access under mild conditions; calipash and bone, however, are rich in highly cross-linked insoluble collagen and typically require combined physical pretreatments to improve hydrolysis efficiency. Currently, the commercial enzymes used for preparing soft-shelled turtle peptides mainly include trypsin, pepsin, alkaline protease, flavour protease, and neutral protease [33]. As the cleavage sites of different types of proteases vary, the hydrolysis products generated following enzyme-substrate interaction also differ in terms of polypeptide molecular weight distribution, amino acid sequence and biological activity. Consequently, in actual production, either a single-enzyme hydrolysis method or a combined hydrolysis method using two or more proteases is typically employed. Furthermore, the yield of soft-shelled turtle peptides is often influenced by various factors, including hydrolysis temperature, hydrolysis time, enzyme dosage, and the pH of the reaction system [31]. Among these, the selection of protease types and the optimization of hydrolysis time are the key factors affecting peptide yield and activity. Nevertheless, the reliance on specific commercial proteases inherently limits the diversity of releasable peptide sequences, and prolonged hydrolysis may generate excessive short-chain peptides that diminish bioactivity while complicating downstream purification.

Fermentation is a biosynthetic technique that utilizes the metabolic and enzymatic systems of microorganisms to convert animal and plant proteins, microbial proteins or nitrogen-containing substrates into small-molecule peptides [34]. Compared with enzymatic hydrolysis, fermentation offers advantages such as lower costs, milder conditions, and the ability to simultaneously remove odours and enhance flavour [35]. This method is particularly advantageous for processing recalcitrant tissues like calipash and cartilage, where the complex microbial enzyme system can partially degrade the dense collagen matrix while producing flavour-enhancing metabolites that mask off-odors. The microbial strains currently used in soft-shelled turtle peptide fermentation primarily include *Bacillus subtilis* [36], lactic acid bacteria [37], and yeast [38]. During fermentation, the proteolytic enzymes secreted by the microorganisms gradually degrade macromolecular proteins into small-molecule peptides and free amino acids. Meanwhile, organic acids, alcohols and other substances produced through metabolism improve the product’s flavour. However, fermentation typically requires longer incubation times (24–72 h), which reduces throughput and increases contamination risk; the resulting peptide profile is complex and less predictable, and the necessary removal of microbial biomass post-fermentation may lower overall peptide recovery. Research indicates that by optimising conditions such as the combination of fermentation strains, fermentation temperature, duration and initial pH, the yield and biological activity of soft-shelled turtle peptides can be significantly improved [39]. Soft-shelled turtle peptides prepared via fermentation exhibit good activity in terms of antioxidant and immunomodulatory effects, and are more readily absorbed and utilized by the human body.

Recent emerging technologies have significantly advanced the preparation of soft-shelled turtle peptides, with some methods already directly implemented and others providing critical methodological blueprints from related bioactive peptide research. A prominent strategy involves ultrasound-assisted enzymatic hydrolysis, it significantly enhanced the extraction yield of collagen from soft-shelled turtle calipash and skin by disrupting the extracellular matrix through acoustic cavitation, while maintaining the native structure and improving solubility and antioxidant activity of the resultant peptides [13]. Furthermore, AI-assisted peptide discovery has been actively integrated into soft-shelled turtle peptide research, utilizing machine learning algorithms and bioinformatics tools to analyze complex proteomic data, predict peptide structures and bioactivities, and enable the rapid virtual screening of novel antioxidant and immunomodulatory peptides from soft-shelled turtle hydrolysates [14]. In contrast, microwave-assisted extraction (MAE), while not yet reported specifically for soft-shelled turtle peptide extraction, has been widely applied to other animal and plant proteins, where rapid internal heating via ionic rotation and dipole polarization efficiently releases intracellular peptides; this approach offers a promising rapid-extraction strategy that could be adapted for turtle peptide preparation [40]. Similarly, pulsed electric field (PEF) technology, though not yet applied to turtle peptides, has been successfully used in marine by-products and other animal proteins to induce cell membrane electroporation, providing a non-thermal pretreatment option to improve protein extractability while preserving thermolabile bioactivities [41]. Finally, deep eutectic solvents (DESs), recognized as green and tunable extraction media, have demonstrated exceptional efficiency in extracting bioactive peptides from various food and agricultural by-products; their application to soft-shelled turtle processing could provide a sustainable, low-toxicity alternative to traditional solvents, potentially enhancing both peptide stability and solubility [42].

### 3.2. Separation and Purification of Soft-Shelled Turtle Peptides

The crude peptide product obtained from soft-shelled turtle protein following enzymatic hydrolysis or fermentation is a complex mixture comprising polypeptide components with varying molecular weights, amino acid sequences, charge properties and hydrophobicity, whilst also containing unhydrolysed proteins, free amino acids, salts and other impurities [43]. To further investigate the structural characteristics, structure-activity relationships and biological activity of soft-shelled turtle peptides, it is essential to separate and purify the crude peptide products in order to obtain high-purity, high-activity single peptide segments or active peptide components within a specific molecular weight range. Separation and purification are critical steps in soft-shelled turtle peptide research, directly affecting the accuracy of subsequent structural identification and functional evaluation; The differing properties of peptide segments determine the choice of separation and purification methods (as shown in Table 3). Methods for separating active peptides based on molecular weight include ultrafiltration (UF), nanofiltration (NF) [44] and gel filtration (GFC); for peptide segments with different charges, ion exchange chromatography is used, whilst reverse-phase high-performance liquid chromatography (RP-HPLC) separates peptides based on their hydrophobicity [45].

Membrane separation can rapidly and efficiently separate a crude extract of soft-shelled turtle peptides into components of different molecular weight ranges; however, due to limited resolution, it is difficult to achieve the separation of individual peptides, and it is typically used as a preliminary fractionation method in the first step of separation and purification; gel filtration and ion exchange are often employed as subsequent purification steps, with high-resolution reverse-phase high-performance liquid chromatography (RP-HPLC) ultimately undertaking the purification task. For example, Wang et al. [52] utilised UF membranes to separate mackerel protein hydrolysate (MPH) into three fractions: MPH-I (>10,000 Da), MPH-II (3500–10,000 Da) and MPH-III (<3500 Da). They preliminarily identified MPH-III as the fraction with the highest antioxidant activity, followed by further purification via size-exclusion chromatography using Sephadex G-25 Fine (GE Healthcare Bio-Science AB, Uppsala, Sweden). Finally, the peptide with the longest retention time, MPH-III-2-6, was eluted from a reverse-phase column, indicating that this peptide possessed the highest cellular antioxidant activity.

Following multi-stage separation and purification, the active components obtained require amino acid sequence analysis to determine the relationship between their activity and structure. In peptide identification, mass spectrometry is increasingly widely used for analysing the amino acid sequences of peptides and proteins due to its speed, high-throughput capabilities and high sensitivity. In practical applications, mass spectrometry is often coupled with high-performance liquid chromatography (HPLC), enabling the simultaneous acquisition of peptide retention times, exact molecular weights and fragment ion information in a single analysis, thereby allowing the derivation of complete amino acid sequences [53]. In soft-shelled turtle peptide research, LC-MS/MS has become an indispensable key method for characterising peptide structures.

To bridge the gap between laboratory-scale purification and industrial application, it is essential to evaluate peptide separation techniques not only by resolution, but also by scalability, processing cost, and recovery efficiency. Membrane-based separations, particularly ultrafiltration (UF) and nanofiltration (NF), offer clear advantages for large-scale production of turtle-derived peptides. These methods can process large volumes of hydrolysate continuously, require relatively low capital and operating costs, and achieve peptide recoveries often exceeding 80% for targeted molecular weight fractions [54]. In contrast, chromatographic techniques such as size-exclusion chromatography (SEC) and reversed-phase high-performance liquid chromatography (RP-HPLC), while indispensable for peptide identification and activity-guided purification at the bench scale, suffer from limited loading capacity, high solvent consumption, and low throughput, making them economically prohibitive for bulk production [55,56]. Ion-exchange chromatography (IEC) offers a compromise, but resin regeneration and buffer usage add to processing costs [57]. Recent advances in simulated moving bed (SMB) chromatography and expanded bed adsorption (EBA) have partially addressed the scalability issues of chromatographic methods [58], yet these have not been tested for turtle peptide mixtures. Another industrially feasible option is the use of food-grade macroporous adsorption resins, which can enrich bioactive peptides with moderate resolution at a fraction of the cost of HPLC. Furthermore, isoelectric precipitation or ethanol precipitation can serve as crude fractionation steps, significantly reducing the volume load on subsequent high-resolution techniques and improving overall process economy [59]. In terms of peptide recovery efficiency, it is critical to monitor peptide loss at each step; multi-step purification trains, while achieving high purity, often lead to low cumulative yields, which is unacceptable for commercial nutraceutical production. Therefore, a tiered strategy coupling low-resolution, high-recovery techniques with a single high-resolution step is recommended for scaling up turtle peptide isolation while maintaining an appropriate balance between bioactivity, purity, and cost.

## 4. Functional Activities and Molecular Mechanisms of Soft-Shelled Turtle Peptides

**Figure 3:**
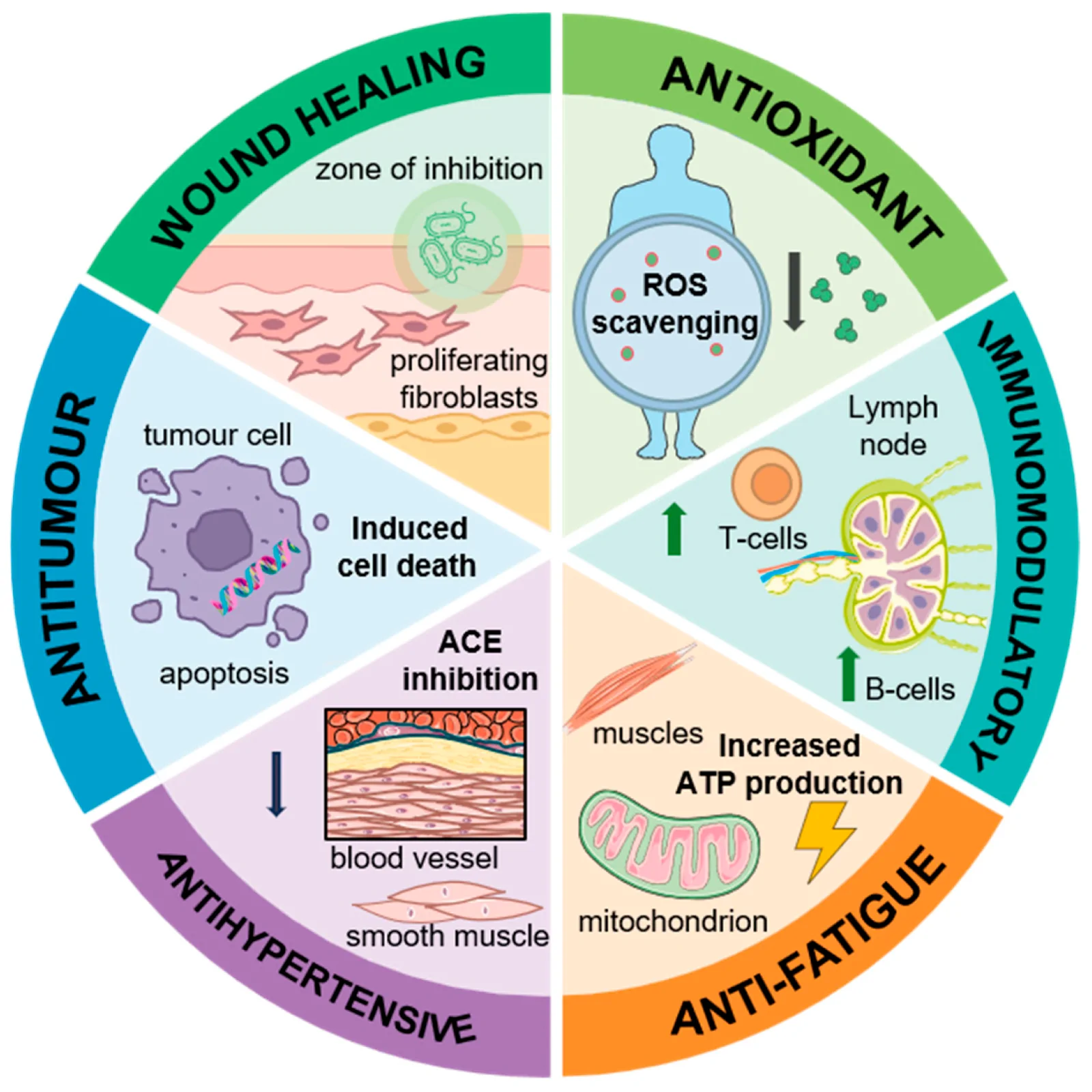
Multiple biological activities of turtle peptide (Green upward arrows indicate an increase in cell quantity or content level, while black downward arrows indicate a decrease or inhibitory effect).

The development of targeted preparation and separation strategies has enabled the acquisition of peptide fractions with defined biochemical characteristics. As a class of small-molecule bioactive peptides derived from animal proteins, soft-shelled turtle peptides exhibit a variety of physiological functions due to their low molecular weight, structural diversity and ease of absorption by the human body. In recent years, scholars both domestically and internationally have conducted systematic research into the bioactivity of soft-shelled turtle peptides, confirming their significant efficacy in antioxidant activity, immune modulation, anti-fatigue effects, blood pressure reduction (ACE inhibition), anti-tumour activity, and the promotion of mineral absorption (Figure 3). These physiological functions do not stem from a single mechanism of action, but rather from a complex network of regulatory mechanisms that modulate multiple signaling pathways and act upon various molecular targets. Accumulating studies have explored the underlying molecular mechanisms of these bioactivities, yet the robustness of supporting evidence varies considerably across different pathways. Most current mechanistic findings are derived from in vitro cell assays and small animal experiments, while systematic clinical validation in humans remains scarce. This section systematically summarizes the functional activities of soft-shelled turtle peptides and their underlying molecular basis, with reference to the current state of experimental support for each proposed pathway.

### 4.1. Antioxidant Activity

Oxidative stress is a major contributing factor to various chronic diseases (such as cardiovascular disease, diabetes and neurodegenerative diseases) [60]. Against this backdrop, food-derived antioxidant peptides have attracted widespread attention due to their advantages, such as high safety and ease of absorption. The antioxidant activity of food peptides is associated with the scavenging of free radicals formed during lipid peroxidation and metal chelation [61]; the structure of antioxidant peptides is characterized by hydrophobic amino acids at the N-terminus and aromatic amino acids in the sequence [62]. Due to their high content of hydrophobic and aromatic amino acids, soft-shelled turtle peptides exhibit excellent free radical scavenging capacity. Studies have shown that soft-shelled turtle peptides can effectively scavenge various free radicals, including 2,2-diphenyl-1-picrylhydrazyl (DPPH·) [63], hydroxyl radicals (·OH) and 2,2′-Azino-bis (3-ethylbenzothiazoline-6-sulfonic acid) radical cation [16]. Research by Zhu et al. [64] indicates that soft-shelled turtle protein peptides with a molecular weight of less than 5 kDa exhibit superior antioxidant activity, suggesting that small-molecule peptides are more effective at scavenging free radicals. At the cellular and animal levels, soft-shelled turtle peptides can significantly increase the activity of superoxide dismutase (SOD), glutathione peroxidase (GSH-Px) and catalase (CAT), whilst simultaneously reducing malondialdehyde (MDA) levels, thereby alleviating lipid peroxidation damage [65].

Accumulating experimental evidence supports the activation of the Nrf2/Keap1 signaling pathway as a core enzymatic mechanism underlying the antioxidant activity of soft-shelled turtle peptides, which enhances the endogenous antioxidant defence system. Numerous studies have demonstrated that nuclear factor E2-related factor 2 (Nrf2) is a key transcription factor regulating the expression of antioxidant enzymes [66], whilst the Kelch-like ECH-associated protein 1 (Keap1) is considered to be an inhibitor of Nrf2-mediated activation of the antioxidant response element (ARE) [67]. Under resting conditions, Nrf2 binds to Keap1 in the cytoplasm and is continuously degraded via the ubiquitin-proteasome pathway, thereby maintaining its expression at low levels. Upon entry into the cell, soft-shelled turtle peptide induces a conformational change or oxidative modification of Keap1, leading to the dissociation of Nrf2 from Keap1 and its translocation to the cell nucleus. Within the nucleus, Nrf2 forms a heterodimer with the small Maf protein, binds to the antioxidant response element (ARE), and initiates the transcription of downstream antioxidant enzyme genes, including HO-1, NQO1, SOD, GSH-Px and CAT [68]. Cellular studies have consistently demonstrated that treatment with soft-shelled turtle peptides markedly enhances Nrf2 nuclear accumulation, significantly upregulates the expression of HO-1, NQO1 and SOD, and reduces the levels of oxidative damage markers such as malondialdehyde (MDA) and protein carbonyl groups [69].

Furthermore, soft-shelled turtle peptides can exert antioxidant effects via non-enzymatic pathways, as verified by in vitro activity assays, such as directly scavenging free radicals and chelating transition metal ions, thereby forming a synergistic effect with the enzymatic antioxidant system mediated by the Nrf2 pathway [12]. These results indicate that soft-shelled turtle peptides can serve as natural antioxidants and be applied in functional foods designed to delay ageing and alleviate diseases associated with oxidative stress.

### 4.2. Immunomodulatory Activity

Soft-shelled turtle peptides exert a bidirectional regulatory effect on the immune system, enhancing immune function in states of immunosuppression whilst suppressing excessive immune responses, thereby demonstrating excellent capacity for regulating immune homeostasis. In terms of non-specific immunity, soft-shelled turtle peptides promote phagocytic activity in macrophages and enhance the cytotoxic capacity of natural killer (NK) cells. Studies have shown that the phagocytic index of macrophages treated with soft-shelled turtle peptides increased significantly, and the secretion of cytokines such as nitric oxide (NO), tumour necrosis factor-α (TNF-α) and interleukin-6 (IL-6) increased markedly [70]. In terms of specific immunity, soft-shelled turtle peptides promote the proliferation of splenic lymphocytes, elevate serum levels of immunoglobulins IgG, IgA and IgM, and enhance humoral immune responses [71]. Furthermore, soft-shelled turtle peptides exert an immunomodulatory effect on the intestinal mucosa by regulating the structure and function of the gut microbiota.

The immunomodulatory effects of soft-shelled turtle peptides are mediated through multiple complementary pathways, with the TLR4/NF-κB signaling cascade, macrophage functional modulation, and intestinal mucosal immunity being the most extensively investigated directions. These regulatory pathways are supported by varying levels of experimental evidence: some core conclusions have been validated by dedicated cellular and animal assays, while others remain putative deductions based on current phenotypic observations and homologous pathway paradigms. (1) TLR4/NF-κB signaling pathway: Toll-like receptor 4 (TLR4) is a key receptor for recognising pathogen-associated molecular patterns, and its activation initiates downstream signaling cascades. Cellular studies have consistently demonstrated that soft-shelled turtle peptide treatment markedly modulates NF-κB p65 nuclear translocation in macrophages and regulates the secretion of pro-inflammatory cytokines including TNF-α and IL-6, confirming the involvement of the NF-κB pathway in the immunomodulatory activity of turtle peptides. Based on the canonical TLR4 signaling paradigm and molecular docking simulations, this activation is proposed to be initiated by the binding of turtle peptides to the TLR4/MD2 complex on the macrophage surface, which recruits the adaptor protein MyD88 via its intracellular TIR domain and sequentially activates downstream interleukin-1 receptor-associated kinase (IRAK) and tumour necrosis factor receptor-associated factor 6 (TRAF6), thereby forming a signaling complex to activate the IKK kinase. Activated IKK phosphorylates the inhibitory protein IκBα, leading to its degradation via the ubiquitin–proteasome system and the subsequent release of NF-κB, which then translocates into the nucleus to initiate the transcription of genes encoding inflammatory cytokines (such as TNF-α, IL-1β, and IL-6) and other immune-related genes. Studies have shown that in macrophages treated with soft-shelled turtle peptide, NF-κB p65 nuclear translocation is markedly enhanced, and the secretion of TNF-α and IL-6 is significantly increased [70]. (2) Macrophage polarization: The potential of soft-shelled turtle peptides to regulate macrophage polarization has been proposed in recent studies, but direct and comprehensive experimental evidence remains limited. In vitro functional assays have confirmed that turtle peptides enhance the phagocytic activity of macrophages and promote the production of nitric oxide (NO) and reactive oxygen species (ROS), which are characteristic functional phenotypes of classically activated (M1) macrophages [72]. Building on these observations, it is hypothesized that turtle peptides may drive macrophage polarization towards the M1 phenotype, thereby strengthening anti-pathogenic defence and antigen presentation capacity. It has also been suggested that active peptides may maintain Th1/Th2 immune balance by modulating cytokine secretion to prevent excessive immune activation, but this claim requires further targeted in vivo validation. (3) Regulation of intestinal mucosal immunity: In vivo animal studies have verified that soft-shelled turtle peptides can indirectly influence intestinal mucosal immunity by modulating the structure of the gut microbiota, which constitutes the experimentally supported foundation for their regulatory effects on intestinal mucosal immunity. Specifically, turtle peptide intervention has been shown to upregulate the expression of tight junction proteins (such as Occludin, Claudin-1 and ZO-1), promote mucosal repair, reduce intestinal permeability, and prevent the translocation of bacteria and endotoxins. With respect to microbial regulation, turtle peptides increase the abundance of beneficial bacteria such as *Lactobacillus* and *Bifidobacterium*, reducing the proportion of opportunistic pathogens, promoting the production of short-chain fatty acids (SCFAs), and maintaining intestinal immune homeostasis [73]. It is further postulated that turtle peptides may modulate local intestinal immune responses by influencing the activity and cytokine secretion of intestinal intraepithelial lymphocytes, and that this gut-immune axis regulatory mechanism may provide new perspectives for the application of turtle peptides in immunomodulatory functional foods. Nevertheless, the specific effects on intestinal immune cell subsets and the complete mechanistic chain of the gut-immune axis have yet to be systematically characterized.

### 4.3. Anti-Fatigue Activity

Anti-fatigue activity is one of the key functions of soft-shelled turtle peptides, which show potential applications in high-intensity exercise recovery, physical exhaustion, and chronic fatigue syndrome. Animal studies have shown that bioactive peptides can significantly prolong the duration of weight-bearing swimming in mice, reduce post-exercise blood lactate and blood urea nitrogen levels, and increase liver and muscle glycogen stores, indicating their ability to delay the onset of exercise-induced fatigue and promote recovery from fatigue [74]. Furthermore, serum creatine kinase (CK) and lactate dehydrogenase (LDH) serve as important physiological indicators frequently used to assess muscle damage following intense exercise [75]. Soft-shelled turtle peptides can reduce the release of CK and LDH post-exercise, suggesting they exert a protective effect against exercise-induced skeletal muscle damage [76].

The anti-fatigue effects of soft-shelled turtle peptides are closely associated with their antioxidant properties and energy metabolism regulatory activities, with two major mechanistic pathways proposed based on current experimental evidence. Regarding antioxidant protection, it has been verified that soft-shelled turtle peptides enhance the activities of antioxidant enzymes in skeletal muscle and liver tissue and mitigate exercise-induced oxidative injury to preserve mitochondrial function. These protective effects are proposed to be mediated through activation of the Nrf2/Keap1 signaling cascade, which aligns with the well-documented antioxidant mechanism of turtle peptides at the cellular level [12]. In terms of energy metabolism regulation, biochemical assays have confirmed that soft-shelled turtle peptides can upregulate mitochondrial SDH and COX activity, enhance mitochondrial respiratory chain function, and promote ATP production; they increase liver and muscle glycogen stores, reduce post-exercise blood lactate and blood urea nitrogen levels, and delay glycogen depletion; they also lower post-exercise serum CK and LDH levels, thereby protecting skeletal muscle cell membrane integrity [77]. Building on these phenotypic findings, it is inferred that turtle peptides may enhance mitochondrial respiratory chain function and promote ATP production, thereby improving energy supply efficiency during exercise. Collectively, the anti-fatigue activity of soft-shelled turtle peptides is considered to stem from the synergistic action of antioxidant defense and energy metabolism modulation, providing preliminary experimental support for their development as active ingredients in sports nutrition products and anti-fatigue functional foods.

### 4.4. Antihypertensive Activity (ACE Inhibitory Activity)

Angiotensin-converting enzyme (ACE) is a key enzyme in the regulation of blood pressure. It contributes to blood pressure by converting angiotensin I (Ang I) into angiotensin II (Ang II), which has a potent vasoconstrictive effect, and by inactivating the vasodilator bradykinin [78]. ACE-inhibiting peptides have become an important research focus in research on antihypertensive functional foods. Pujiastuti et al. [79] employed orthogonal bioassay-guided fractionation (RP and SCX) to effectively screen for and identify ACE-inhibitory (ACE I) peptides in soft-shelled turtle peptides. Following oral administration of these peptides to spontaneously hypertensive rats, systolic blood pressure (SBP) decreased significantly within 24 h, demonstrating strong antihypertensive potential. Compared with chemically synthesised antihypertensive drugs, soft-shelled turtle peptides are of natural origin and have few side effects, and can serve as functional food ingredients for adjunctive intervention in hypertension.

Multiple modes of action have been documented for food-derived ACE inhibitory peptides, and the inhibitory patterns of soft-shelled turtle-derived peptides have been partially validated via enzymatic kinetic assays. Competitive inhibition is the most thoroughly verified mode for turtle-derived ACE peptides: enzymatic kinetic analysis with Lineweaver-Burk plots has confirmed that purified turtle egg-derived ACE inhibitory peptides act as competitive inhibitors, which bind to the ACE active site and compete with the substrate angiotensin I (Ang I) for catalytic pocket occupancy, thereby reducing angiotensin II (Ang II) generation [80]. Non-competitive inhibition represents another well-validated inhibitory mode among food-derived bioactive peptides, in which peptides interact with allosteric non-active sites of ACE to induce conformational changes and diminish catalytic efficiency [81]. However, this mode has not been directly confirmed for soft-shelled turtle peptides via targeted kinetic assays, and remains a putative mechanism based on findings from other food-derived peptide systems. Metal ion chelation represents a well-recognized inhibitory mechanism for food-derived ACE peptides, as the catalytic activity of ACE is strictly dependent on the Zn^2+^ cofactor located within the conserved HEXXH motif of the active center [82]. Structurally, inhibitory peptides can chelate the active-site Zn^2+^ via the side-chain groups of specific residues, typically histidine, glutamic and aspartic, thereby disrupting the catalytic zinc coordination environment and blocking enzymatic activity. For soft-shelled turtle-derived ACE inhibitory peptides, this zinc-chelating mode has received preliminary support from molecular docking simulations, which demonstrate that active peptides can form stable coordinate interactions with the Zn^2+^ ion in the ACE catalytic pocket [80].

From a structural perspective, these inhibitory modes are intrinsically linked to the amino acid composition of peptide sequences. Structure-activity studies on food-derived ACE inhibitory peptides consistently demonstrate that C-terminal hydrophobic or aromatic amino acids enhance inhibitory potency by mediating stable interactions with ACE hydrophobic pockets and the catalytic Zn^2+^ [83]. Soft-shelled turtle-derived ACE inhibitory peptides conform to this well-established structural rule, which underpins their binding affinity and inhibitory efficacy.

Notably, current studies on ACE-inhibitory peptides from soft-shelled turtles are still confined to in vitro enzymatic evaluation. Systematic research covering gastrointestinal stability, oral bioavailability, pharmacokinetic behaviour, toxicological safety and clinical relevance remaining largely unavailable, posing a prominent barrier to practical translation. As generally acknowledged for food-derived bioactive peptides, gastrointestinal degradation and low intestinal absorption are universal obstacles limiting oral bioavailability [84], and preliminary findings on other turtle egg-derived bioactive peptides have verified partial peptide hydrolysis during simulated gastrointestinal digestion [85], indicating comparable stability risks for turtle ACE-inhibitory peptides.

### 4.5. Antitumour Activity

Some small-molecule soft-shelled turtle peptides have been shown to inhibit tumour cell proliferation and induce apoptosis in vitro, providing potential bioactive components for adjuvant cancer therapy. Wang et al. [86] used neutral proteases to digest soft-shelled turtle alkaline-soluble protein; the small-molecule peptides isolated from this process exhibited significant proliferation-inhibitory effects on both HepG2 liver cancer cells and MCF-7 breast cancer cells, with a half-maximal inhibitory concentration (IC_50_) of 32.26 μg/mL. Morphological observations showed that the peptide could induce typical apoptotic features in tumour cells, such as cell shrinkage, nuclear condensation and the formation of apoptotic bodies.

The antitumour activity of soft-shelled turtle peptides is mediated through multiple signaling pathways, with varying degrees of supporting experimental evidence. The induction of mitochondrial apoptosis represents the most well-validated direct antitumour mechanism. In vitro cancer cell studies have confirmed that bioactive peptides can upregulate the expression of the pro-apoptotic protein Bax, downregulate the expression of the anti-apoptotic protein Bcl-2, activate the mitochondrial apoptotic pathway, and consequently activate Caspase-3 and Caspase-9, thereby inducing cell apoptosis [87]. In addition, peptide treatment has been shown to significantly upregulate tumour-suppressor miRNAs including miR-375 in cancer cells, suggesting that these peptides may limit tumour cell proliferation and invasion via the Hippo pathway, whilst simultaneously promoting apoptosis and inhibiting tumour growth via the FoxO pathway, thereby exerting their direct anti-cancer effects [88]. Furthermore, soft-shelled turtle peptides are hypothesized to exert indirect antitumour effects by enhancing immune function, though targeted validation of this immunomodulation-mediated pathway remains scarce.

Currently, research into the antitumour activity of soft-shelled turtle peptides is primarily concentrated at the in vitro cellular level. Critical translational data including gastrointestinal stability, systemic bioavailability, pharmacokinetic profiles, long-term toxicity and clinical efficacy are completely absent. These research gaps are commonly observed across most food-derived anticancer peptides at the exploratory stage. In the future, systematic animal experiments and preclinical studies need to be conducted to provide scientific evidence for their application in adjuvant cancer therapy.

### 4.6. Other Functional Activities

In addition to the primary functions mentioned above, soft-shelled turtle peptides have also been reported to possess various biological activities, including antibacterial, anti-inflammatory and wound-healing properties, although the evidence base for these activities remains comparatively limited. With respect to antibacterial activity, in vitro studies have demonstrated that certain turtle peptide fragments exhibit inhibitory effects against common pathogenic bacteria including *Staphylococcus aureus*, *Escherichia coli* [89] and *Pseudomonas aeruginosa* [90], suggesting their potential application in food preservation and anti-infective fields. However, the minimum inhibitory concentrations (MICs) and the specific peptide sequences responsible for these effects have not been systematically characterized. Regarding mineral-binding capacity, in vitro assays have shown that soft-shelled turtle peptides can form soluble complexes with calcium ions, potentially preventing calcium precipitation in the neutral or slightly alkaline environment of the intestine, thereby enhancing calcium bioavailability [91]. These mineral absorption-promoting properties make soft-shelled turtle peptides suitable for the development of functional foods for calcium and iron supplementation, particularly for special populations such as children, the elderly and pregnant women.

### 4.7. Medicinal Potential and Clinical Translation

Soft-shelled turtle peptides exhibit pharmacological potential that extends across multiple therapeutic areas. For cardiovascular health, the ACE-inhibitory activity demonstrated in vitro has been substantiated by in vivo antihypertensive effects in spontaneously hypertensive rats for select muscle and egg white peptides [18,80]. In the context of immune-mediated disorders, turtle peptides have been shown to ameliorate intestinal mucosal immunity in immunosuppressed mice through the AHR/IL-22/STAT3/IL-6 pathway [71] and to attenuate DSS-induced ulcerative colitis by inhibiting inflammation and modulating the gut microbiota [73]. An antiproliferative peptide purified from turtle protein hydrolysates has further demonstrated activity against cancer cells, suggesting potential in adjunctive oncology [86]. Despite these diverse bioactivities, the current evidence base remains overwhelmingly preclinical. The oral bioavailability of intact peptides is severely limited by gastrointestinal degradation, and human pharmacokinetic, dose–response, and long-term safety data are entirely absent [92]. The lack of standardized production protocols and pharmaceutical-grade purity further impedes clinical translation. Future work must integrate well-designed human trials, rigorous pharmacokinetic evaluation, and scalable manufacturing to establish the true clinical viability of turtle-derived peptides.

## 5. Conclusions and Outlook

The Chinese soft-shelled turtle (*Pelodiscus sinensis*) is an internationally significant model for bioactive peptide research owing to its unique evolutionary position, diverse tissue biochemistry, and long-documented health-promoting effects, with its large-scale farming across East Asia and introduced populations worldwide further underscoring its global relevance. This review provides a comprehensive overview of the bioactivities of soft-shelled turtle peptides and their underlying molecular mechanisms and systematically evaluates the feasibility of using various turtle parts as peptide sources. Despite these promising prospects, several challenges remain: off-flavors limit direct food application, in vivo efficacy remains largely unverified, and systematic safety assessments are still lacking. Future research should prioritize targeted deodorization technologies, in vivo mechanistic studies, and comprehensive toxicological evaluations. Additionally, advanced extraction strategies such as irradiation-assisted methods and green solvents deserve exploration to improve extraction efficiency and sustainability. Addressing these issues will be essential to bridge the gap between laboratory findings and industrial applications and to establish the corresponding regulatory framework for commercialization.

## Figures and Tables

**Table 1:** Research progress on hydrolyzed peptides from soft-shelled turtles and their by-products.

Source	Peptide Sequence	Hydrolysis Method	Biological Activities	References
Soft-shelled turtle muscle	GKIYEQCELA, NCA, EDYGA, CTA, CGIECSELLKA, DLLCIV, WTKYCKGKDV. Molecular weight < 3000 Da	Chopped soft-shelled turtle muscle was mixed with distilled water in a 1:4 ratio and homogenized for 10 min using a homogenizer. The mixture was adjusted to pH 7.0, Protamex was added at an enzyme-to-substrate ratio of 3500 U/g, and the mixture was incubated at 50 °C for 4 h.	The purified peptide fragment shows DPPH radical scavenging activity. The representative peptide EDYGA activates the Nrf2-ARE antioxidant pathway via Keap1 downregulation and Nrf2 stabilization. The corresponding hydrolysate also exhibits concentration-dependent hydroxyl radical scavenging capacity, reducing power and Fe^2+^ chelating activity.	[9,12]
Soft-shell turtle calipash	Acidic collagen	Prior to extraction, the sample solution was subjected to ultrasonic treatment. The pre-treated calipash particles were suspended in 0.5 mol/L acetic acid at a solid-to-liquid ratio of 1:20 (g/mL), stirred and extracted for 24 h, centrifuged to collect the supernatant, and salt-precipitated by adding NaCl. The precipitate was redissolved in acetic acid, the salt was removed by dialysis, and finally, the ASC powder was obtained by freeze-drying	Collagen itself has weak antioxidant activity, but after enzymatic hydrolysis in the gastrointestinal tract, it produces small-molecule bioactive peptides, thereby exhibiting antioxidant activity	[13]
Soft-shelled turtle bones and shell	Collagen-derived peptides (GPPGVPGPL, TSLPVPAPV, QICVCDS, DVWK, IIEY, APMDVG), molecular weights ranging from 536.6 to 1016.5 Da	For turtle bones, virtual hydrolysis of bone collagen sequences was performed using the “papain + fig protease” combination screened via the BIOPEP-UWM database. For turtle shells, after washing to remove impurities, drying and grinding, crude protein was extracted via combined enzymatic hydrolysis with pepsin and trypsin	The shell-derived hydrolysate possesses anti-hepatic fibrosis activity (IC_50_: 80.13–190.0 μM); bone-derived peptides show excellent physicochemical properties and pharmacokinetic characteristics.	[8,14]
Soft-shelled turtle offal	Collagen	Enzymatic hydrolysis and extraction were performed using papain; the optimal conditions were: temperature 32 °C, enzyme concentration 4.0 mg/mL, solid-to-liquid ratio 1:35, and extraction time 12 h	-	[15]
Soft-shelled turtle eggs	HNKPEVEVR (1106.58 Da), ARDASVLK (858.49 Da), SGTLLHK (754.43 Da)	The soft-shelled turtle egg raw material was first pretreated to obtain total protein. Papain was subsequently added at an enzyme-to-substrate ratio of 1:50 (*w*/*w*), and the hydrolysis reaction was carried out at pH 7.0 and 50 °C for 4 h. After the reaction was terminated, the mixture was cooled and centrifuged (10,000× *g*, 15 min), and the resulting supernatant was collected as the soft-shelled turtle egg protein hydrolysate.	which exhibits significant α-glucosidase inhibitory activity and free radical scavenging capacity	[16]

**Table 2:** Comparison of bioactive peptide profiles derived from *Pelodiscus sinensis* and *Chinemys reevesii*.

Comparison Dimension	*Pelodiscus sinensis*	*Chinemys reevesii*
Tissue/Material Studied	Muscle, egg, calipash, shell, offal and bone	Muscle
Commonly Used Proteases	Papain, Subtilisin, etc.	Papain
Peptide Molecular Weight Distribution	Peptides < 1000 Da account for 91.38% [9]	89.23% of peptides are < 1000 Da [3]
Number of Identified Monomeric Peptides	Over 30 (from multiple tissues) [12,18]	About 10 [3]
Examples of Representative Peptides	ARDASVLK, HNKPEVEVR, EDYGA, LVDGVIIR, etc.	WGDAGAE, LSEE, HELE, TVEE, etc.
Main Bioactivities	Antioxidant, ACE inhibitory, immunomodulatory	Antioxidant, Anti-aging (activation of the Nrf2/Keap1 pathway), metal-chelating
Activity Comparison	DPPH (77.45%), ACE inhibitory (IC_50_: 41.33 μM) [12,18]	DPPH (75.89%), iron chelating (63.25%) [31]

**Table 3:** Characteristics of different separation and purification techniques for soft-shelled turtle peptides.

Separation and Purification Method		Mechanism	Separation Method	Advantages	Disadvantages	Reference
Membrane Separation	Microfiltration (MF)	Achieves physical separation by exploiting the difference between the membrane pore size and the size of the target molecules. When the feed solution flows across the membrane surface under pressure, macromolecules larger than the membrane pores are retained on the upstream side of the membrane, whilst small molecules or solvents smaller than the membrane pores pass through the pores and collect on the downstream side	Typically specified by pore size: 0.1–10 μm; MWCO (Da) > 1000 kDa	High processing efficiency, strong resistance to fouling and low susceptibility to blockage, providing UF/NF with a clean feed solution that is less prone to clogging	It can only retain suspended solids, bacteria and large colloidal particles; it cannot retain dissolved molecules	[46]
	Ultrafiltration (UF)		MWCO (Da): 1000 Da–1000 kDa	Enables precise separation based on the correlation between soft-shelled turtle peptide activity and molecular weight; simple to operate, low-cost, energy-efficient and environmentally friendly; carried out at room temperature to avoid high-temperature degradation of active peptides, effectively preserving their activity	Prone to contamination by adsorption of macromolecules such as proteins, colloids and polysaccharides; unable to separate different small molecules	[47]
Membrane Separation	Nanofiltration (NF)		MWCO (Da): 200 Da–1000 Da	The separation process requires no chemical reagents, no heating and no phase change. It achieves the loss-free concentration of active peptides whilst removing salts from the enzymatic hydrolysate	Research into the design of nanofiltration membrane materials, as well as membrane performance and structure, is not yet fully developed	[48]
Chromatography	Gel Filtration Chromatography (GFC)	Molecular sieve effect of porous gel packing. Depending on differences in molecular size, small-molecule peptides enter the internal channels, have a long elution path, and elute later; large-molecule peptides are excluded from the channels and elute first	Classified by molecular weight, the most commonly used Sephadex series—G-10 (<700 Da), G-15, and G-25 (1–5 kDa)—allow for the selection of different gel types based on the molecular weight range of soft-shelled turtle peptides	The conditions are mild (using physiological buffer systems), no organic solvents are employed, and the antioxidant and other biological activities of the peptides are not compromised; this method is the most widely used in the purification of soft-shelled turtle peptides	Separation is slow, and it is difficult to separate peptide fragments with similar molecular weights; resolution is far lower than that of HPLC, and it is typically used as an intermediate purification step	[49]
Chromatography	Ion Exchange Chromatography (IEC)	Electrostatic interactions. Based on the nature (positive/negative) and intensity of the net charge of peptide fragments under specific pH conditions, charged peptide fragments bind to a stationary-phase ion exchange resin with the opposite charge; selective elution is achieved by gradually increasing the salt concentration or altering the pH gradient	Cation exchange (SCX, pH < pI) and anion exchange (SAX, pH > pI)	High binding capacity, superior resolution compared to GFC, and moderate cost; the separation mechanism is orthogonal to that of GFC (charge vs. size), and when used in combination, purification efficiency can be significantly improved	The pH and ionic strength need to be optimised; neutral peptides are difficult to separate effectively; high-salt eluents require subsequent desalting	[50]
	Reverse-phase high-performance liquid chromatography (RP-HPLC)	Hydrophobic interactions. Based on differences in peptide segment hydrophobicity, reversible adsorption occurs between non-polar stationary phases (C18/C8 alkyl chains, etc.) and the hydrophobic residues of peptide segments in the mobile phase, with elution achieved by increasing the organic solvent gradient.	Separation is based on hydrophobic differences; commonly uses C18 columns with a water/acetonitrile (containing 0.1% TFA) gradient elution, and a detection wavelength of 214–220 nm	This method offers extremely high resolution, good reproducibility, and is suitable for automated operation; when coupled online with ESI-MS, it enables simultaneous peptide identification. It serves as the final step in the fine purification of soft-shelled turtle peptides	Small sample capacity, high consumption of organic solvents, and relatively high costs; separation of highly polar or highly hydrophobic peptides is less effective	[51]

## Data Availability

No new data were created or analyzed in this study. Data sharing is not applicable to this article.
